# Context‐dependent responses of food‐hoarding to competitors in *Apodemus peninsulae*: implications for coexistence among asymmetrical species

**DOI:** 10.1111/1749-4877.12425

**Published:** 2020-03-01

**Authors:** Hongyu NIU, Jie ZHANG, Zhiyong WANG, Guangchuan HUANG, Chao PENG, Hongmao ZHANG

**Affiliations:** ^1^ Institute of Ecology and Evolution, School of Life Sciences Central China Normal University Wuhan China

**Keywords:** asymmetrical food competition, behavioral plasticity, food‐hoarding, species coexistence, sympatric rodents

## Abstract

Superior species may have distinct advantages over subordinates within asymmetrical interactions among sympatric animals. However, exactly how the subordinate species coexists with superior species is unknown. In the forests west of Beijing City, intense asymmetrical interactions of food competition exist among granivorous rodents (e.g. *Apodemus peninsulae, Niviventer confucianus, Sciurotamias davidianus* and *Tscherskia triton*) that have broadly overlapping habitats and diets but have varied body size (range 15–300 g), hoarding habits (scatter *vs* larder) and/or daily rhythm (diurnal *vs* nocturnal). The smallest rodent, *A. peninsulae*, which typically faces high competitive pressure from larger rodents, is an ideal model to explore how subordinate species coexist with superior species. Under semi‐natural enclosure conditions, we tested responses of seed‐hoarding behavior in *A. peninsulae* to intraspecific and interspecific competitors in the situations of pre‐competition (without competitor), competition (with competitor) and post‐competition (competitor removed). The results showed that for *A. peninsulae*, the intensity of larder‐hoarding increased and the intensity of scatter‐hoarding declined in the presence of intraspecifics and *S. davidianus*, whereas *A. peninsulae* ceased foraging and hoarding in the presence of *N. confucianus* and *T. triton. A. peninsulae* reduced intensity of hoarding outside the nest and moved more seeds into the nest for larder‐hoarding under competition from intraspecific individuals and *S. davidianus*. In most cases, the experimental animals could recover to their original state of pre‐competition when competitors were removed. These results suggest that subordinate species contextually regulate their food‐hoarding strategies according to different competitors, promoting species coexistence among sympatric animals that have asymmetrical food competition.

## INTRODUCTION

Food-hoarding is an evolutionary adaption that has evolved in some animals (e.g. rodents and birds) in response to spatial and temporal variation in the availability of resources (Vander Wall 1990). Animals often store food during a food-rich period (e.g. in autumn) and rely on these foods for survival and/or reproduction in the following periods of food scarcity (e.g. in winter and early spring), thereby increasing fitness of survival and reproduction (Vander Wall 1990). The food-hoarding spectrum varies from larder-hoarding (concentrating food items in a few locations) to scatter-hoarding (with multiple small caches) under different conditions (Vander Wall 1990; Dally *et al.* 2006).

A major requisite for evolution of food-hoarding behavior is that hoarders have to gain an advantage from hoarding events thereby animals strive to protect stores and retrieve more caches during the period of food scarcity than native pilferers (Vander Wall 1990; Gu *et al.* 2017). As an important consequence of food loss, food pilferage by intraspecific and interspecific competitors threatens the survival and the reproductive capacity of hoarders when food is scarce (Wauters *et al.* 1995; Vander Wall & Jenkins 2003; Gerhardt 2005). Evidently, food hoarders are sensitive to competitors and have evolved an array of strategies to reduce or prevent the risk of pilferage posed by competitors (reviewed by Vander Wall & Jenkins 2003; Dally *et al.* 2006; Grodzinski & Clayton 2010).

Food hoarders can adopt one or more of the following strategies to compete for food and/or minimize and compensate for food loss. First, food hoarders can limit or invalidate the information that a potential pilferer has gathered (MacDonald 1976; Dally *et al.* 2006; Grodzinski & Clayton 2010). Some hoarders hoard foods in secret locations far away from the sources (Galvez *et al.* 2009; Zhang *et al.* 2014a; Steele *et al.* 2014, 2015), recache items to invalidate pilferers' sensory information (Dally *et al.* 2005, 2006), use misinformation (e.g. empty caches) to confuse potential pilferers (Bugnyar & Kotrschal 2004; Steele *et al.* 2008) and/or conceal auditory information from potential pilferers (Stulp *et al.* 2009). Second, animals can tolerate a partial loss of their total caches because they hoard more food items than they need or steal caches from other hoarders to compensate for the loss (Vander Wall & Jenkins 2003). Third, animals compensate for food loss by increasing eating and/or hoarding intensity (Huang *et al.* 2011; Zhang *et al.* 2011; Luo *et al.* 2014). Finally, animals aggressively prevent competitors from accessing cached sites (Clarke & Kramer 1994), or shift from scatter-hoarding to larder-hoarding to facilitate cache protection or vice versa to avoid a complete loss (Jenkins *et al.* 1995; Preston & Jacobs 2001; Huang *et al.* 2011; Zhang *et al.* 2011; Luo *et al.* 2014). These strategies are not mutually exclusive and are context-dependent for a given species. While comprehensive, most of the above responses and adaptations have been described from patterns of intraspecific competition/pilferage, and more research is needed to study food competition between interspecific hoarders (but see Leaver & Daly 2001; Thayer & Vander Wall 2005; Vander Wall *et al.* 2009; Penner & Devenport 2011; Zhang *et al.* 2013b; Dittel *et al.* 2017).

Interactions in food-hoarding between sympatric rodents are associated with species coexistence (Leaver & Daly 2001; Price & Mittler 2003, 2006; Vander Wall *et al.* 2009; Penner & Devenport 2011; Luo *et al.* 2014; Dittel *et al.* 2017; Gu *et al.* 2017; Wang *et al.* 2018). Due to differential capabilities of food competition (e.g. body size), sympatric species usually exhibit asymmetrical interactions in that some species (superior species hereafter) may have distinct advantages over others (subordinate species hereafter) (Vander Wall *et al.* 2009; Penner & Devenport 2011; Zhang *et al.* 2014b; Dittel *et al.* 2017). Asymmetrical competition in food-hoarding and pilferage has been observed to occur between intraspecific individuals that have different social statuses (e.g. Zhang *et al.* 2011; Huang *et al.* 2019) and sympatric species that have different body sizes, hoarding habits, daily rhythms and/or diets (e.g. Vander Wall *et al.* 2009; Penner & Devenport 2011; Zhang *et al.* 2014b; Dittel *et al.* 2017; Gu *et al.* 2017). In the case of asymmetrical interactions, context-dependent regulation of hoarding strategy is critically important for survival of the subordinate species that endures heavy cache losses, and coexistence of sympatric species that share similar habitats and diets. However, this behavioral plasticity of food-hoarding has received little attention.

At our study site in the Donglingshan mountains west of Beijing City, there exists a rich community of granivorous rodents [e.g. *Apodemus peninsulae* (Thomas, 1907), *Niviventer confucianus* (Milne-Edwards, 1871), *Sciurotamias davidianus* (Milne-Edwards, 1867), *Tscherskia triton* (de Winton, 1899), *Apodemus agrarius* (Pallas, 1771) and *Eutamias sibiricus* (Laxmann, 1769)] that have broadly overlapping habitats (e.g. secondary forests, shrublands and abandoned farmlands) and diets (e.g. seeds of *Quercus wutaishanica*, *Armeniaca sibirica*, *Juglans mandshurica* and *Amygdalus davidiana*) but have varied body size (range 15–300 g), hoarding habits (scatter *vs* larder) and/or daily rhythms (diurnal *vs* nocturnal) (Table 1) (Li *et al.* 2004; Zhang & Zhang 2008; Zhang *et al.* 2015). These rodent species not only compete for temporally limited plant seeds of the parent trees during the seedfall periods (summer and autumn), but also compete for caches over longer periods after seedfall (winter and early spring), which is essential for their survival during hard winters and reproduction in the following spring (Zhang *et al.* 2015, 2017). These rodent species have adapted an array of strategies to compete for seed resources before hoarding and protect/pilfer caches after hoarding (Huang *et al.* 2011; Zhang *et al.* 2011, 2014a,b; Luo *et al.* 2014). For example, *A. peninsulae*, *N. confucianus* and *S. davidianus* increase hoarding intensity even at times when their stores are repeatedly and completely lost (Huang *et al.* 2011; Luo *et al.* 2014). *S. davidianus* first hoards seeds around seed stations to rapidly sequestrate resources and subsequently transfers these seeds to more secure places (e.g. home range) to facilitate protection (Zhang *et al.* 2014a). *E. sibiricus* can use auditory and visual cues of intraspecific hoarders to pilfer caches of others (Niu *et al.* 2019). Besides increasing hoarding intensity, *A. peninsulae* shifts from larder-hoarding to scatter-hoarding in the presence of intraspecific individuals and vice versa when interspecific competitors (*N. confucianus*) are present (Zhang *et al.* 2011). These interactions of hoarding and pilferage between sympatric species are associated with species coexistence, but interaction networks have not been established at the community level.

The difference in body size, caching and pilferage ability between the rodent species at our study site seems to create an asymmetry in the species interactions, whereby some large species (e.g. *N. confucianus*, *T. triton* and *S. davidianus*) with high competitive ability gain more advantages from food competition and pilferage than the small species (e.g. *A. peninsulae*) (also see Vander Wall *et al.* 2009). Asymmetrical food competition has been observed at the level of pairwise species interactions in the rodent community at our study site. For example, *N. confucianus* (larger-size, larder-hoarding) can pilfer caches from *A. peninsulae* (small-size, larder and scatter-hoarding), but the converse does not occur (Zhang *et al.* 2014b). *A. peninsulae* space their hoarded seeds at a certain depth that can partially prevent pilferage by *N. confucianus* (Zhang *et al.* 2014b). However, asymmetrical food competition among multiple species has not been studied. Under the conditions of asymmetrical food competition, an important but less well known question is how the subordinate species (e.g. *A. peninsulae*) regulate their behavior to maximize fitness during the hoarding events. In the rodent community at our study site, *A. peninsulae* gives us an ideal model to explore this question because this rodent species has the smallest body size but it has to face high risks of competition by sympatric species (e.g. *N. confucianus*, *S. davidianus* and *T. triton*) when it hoards plant seeds.

Under enclosure conditions, we tested the food-hoarding behavioral response of *A. peninsulae* individuals to intraspecific and interspecific competitors when competitors were unavailable (pre-competition), available (competition) and then removed (post-competition). We wanted to know how the experimental animals respond to different competitors and if they can recover to pre-competition status when the competitors are removed. We predicted that food-hoarding behavioral responses of *A. peninsulae* were context-dependent according to different competitors that have different body sizes, food-hoarding habits and/or daily rhythms. If the behavioral plasticity of the subject is caused by competition, then behavioral responses of the experimental animals will recover to the state of pre-competition when competitive pressure is released. Finally, we discussed the implication of behavioral plasticity on survival fitness of subordinate species and the coexistence of sympatric hoarders under asymmetrical competition.

**Table 1** Animals used in the experiments of seed-hoarding behavior of *Apodemus peninsulae* under the mediation of competitors

| Subjects | | Competitors | | | | | | |
|---|---|---|---|---|---|---|---|---|
| Sample size | Body mass (mean ± SE, g) | Species | Sample size | Body mass (mean ± SE, g) | Habitat | Daily rhythm | Hoarding habit | Test time |
| 7♂3♀ | 28.1 ± 0.9 | *Apodemus peninsulae* | 4♂3♀ | 28.7 ± 1.3 | SF, SL, AF | Nocturnal | SH and LH | 2015 |
| 6♂4♀ | 28.7 ± 1.1 | *Niviventer confucianus* | 7♂3♀ | 88.4 ± 4.4 | SF, SL, AF | Nocturnal | LH | 2015 |
| 4♂6♀ | 28.4 ± 1.2 | *Tscheskia triton* | 3♂5♀ | 98.2 ± 6.4 | SL, AF | Nocturnal | LH | 2016 |
| 3♂5♀ | 28.6 ± 1.4 | *Sciurotamias davidianus* | 4♂2♀ | 245.4 ± 10.9 | SF, SL, AF | Diurnal | SH and LH | 2016 |

Habitats are secondary forests (SF), shrublands (SL) and abandoned farmlands (AF). Hoarding habits are scatter hoarders (SH) and larder hoarders (LH).

## MATERIALS AND METHODS

### Study sites and animal handling

The experiments were conducted in the Liyuanling field station, Donglingshan Mountain area, approximately 120 km north-west of Beijing City (40°00′N, 115°30′E, 1100 m a.s.l.). This station has been described in detail in our previous works (Zhang & Zhang 2008; Zhang *et al.* 2013a, 2016).

The rodent species used in this study are common around the station (Table 1) (also see Li *et al.* 2004). All of the experimental animals were captured using live traps (12 × 12 × 25 cm) in 10 plots (2.5 ha, 50–100 m apart) in the secondary forests and shrublands near the field station during the summers of 2015 and 2016 (Table 1). Traps were made of steel wire with an iron sheet attached to the top to protect animals from rain and direct sunlight. Peanuts, local plant seeds (e.g. *Q. wutaishanica* and *A. sibirica*), pieces of cucumber or carrot, and local dry leaves were provided in each trap to ensure the captured animals survived well. Twenty-five traps were placed approximately 5-m apart along each of the 2–3 transects (approximately 100-m long, 20-30-m apart) in each plot. Traps were set in an afternoon (1800-1900 hours) and checked twice per day (at 0600-0700 hours and 1800-1900 hours) during the following 2–4 days. Captured animals were individually covered in the trap using a cloth bag and carefully transferred to the laboratory. Females in pregnancy or lactation, juveniles and other unwanted species were released immediately at the site. Following sex determination, weighing and labeling, animals were individually housed in PVC box (37 × 26 × 17 cm) or wheel cages (100 × 100 × 120 cm, one nest box, 20 × 20 × 20 cm, was attached to one corner, for squirrels) and provided commercial mouse chow (Keao Feed, Beijing, China). Water and nest material (wood chips/cotton) were provided ad libitum. Some local rodent-preferred seeds (e.g. *Q. wutaishanica*, *A. sibirica*, *A. davidiana* and/or *Juglans regia*) and peanuts were provided every week to maintain the animals' natural diets and as a nutritional supplement. Individuals of each species were raised in different rooms with ambient temperature (18–25 °C) and photoperiod (14–16 h of daylight) during the experimental period (August to September in 2015 and 2016). Each animal was acclimatized to the house condition at least 7 d prior to testing. After testing, the experimental animals were released to the sites where they were captured or kept in the laboratory for other experiments. All animals remained healthy until the end of the experiments. All guidelines for animal handling were followed according to Chinese law and the study was permitted by the local government and our institute (permission: SYX-K(é)2015-0052).

### Seed preparation

*A. sibirica* is a common tree/shrub species across northern China. As a dominant species in the secondary forests and shrublands in the study area, *A. sibirica* produces numerous seeds each summer (July to August). *A. sibirica* seeds are ideal for experiments because they are highly preferred by the experimental rodent species for consumption and hoarding (Zhang *et al.* 2011, 2015, 2016). The use of *A. sibirica* seeds is described in our previous works (Huang *et al.* 2011; Zhang *et al.* 2011, 2016).

Experimental seeds were randomly collected from more than 50 *A. sibirica* trees near the field station during the period of natural seedfall (July in 2015 and 2016). All seeds were then mixed to create a single composite sample and kept in the refrigerator (0–4 °C) to maintain their freshness. To facilitate seed-tracking, each experimental seed was marked by a tin-tag: a unique coded tin-tag (30 × 10 mm, 0.1 g weight) was attached to the basal end of the endocarp of each seed using a 3-cm piece of fine steel wire (Zhang & Wang 2001). This method has been widely used to relocate rodent-dispersed seeds under the conditions of enclosures and in the field, although it may delay seed harvest and be used for cues to retrieve/pilfer caches by animals (Xiao *et al.* 2006).

### Enclosures

Four separated semi-nature enclosures (10 × 10 m) used for experiments were constructed in the abandoned farmland at the field station (Fig. 1). The walls of the enclosures were made of bricks (30-cm thick), extended 30 cm below the ground surface and 100 cm above ground. Wire mesh (1 × 1 cm grid) was used as cover for each enclosure to prevent animals from entering or escaping the enclosure (also see Lu & Zhang 2010; Zhang *et al.* 2015). Some grass and branches were scattered on the mesh to simulate canopy cover in the field (approximately 60% coverage). The ground surface of each enclosure was a concrete floor which was covered with 15 cm of sandy soil as a hoarding substrate. Grass (e.g. *Artemisia* spp., *Elymus excelsus* and *Poa* spp.) and shrubs (e.g. young *A. sibirica* and *Ulmus laciniata*) were planted in the enclosures; the plants were 30-80 cm tall with <60% coverage, similar to the vegetation around the enclosures. A nest (a wooden box, 20 × 40 × 20 cm, with cotton as nest material) and a water resource (a plastic plate) for the tested animal were placed in one corner of each enclosure, whereas a competitor was located at the opposite corner (Fig. 1). For tests, an individual competitor was held in a steel-wire cage (30 × 30 × 60 cm) with sufficient food, water and cotton nest material, covered with a plank to shelter the animal from rain and direct sunlight. The tested animal could see, smell and even partially touch the competitor animal. A seed station (0.5 $m^2$) was located at the center of each enclosure. Each enclosure was equally divided into 4 areas according to the level of competitive risk: the quarter closest to competitors indicates high competition, the quarter closest to the subject's nest stands for low competition, while the rest of the area of the enclosure represents medium competition (Fig. 1).

## Experimental procedures

Each experimental animal received 4 consecutive days of testing in each trial: day 1, habituation (without competitor); day 2, pre-competition (pre-control, without competitor); day 3, competition (treatment, with competitor); and day 4, post-competition (post-control, competitor removed). During each trial an experimental animal was introduced into an enclosure between

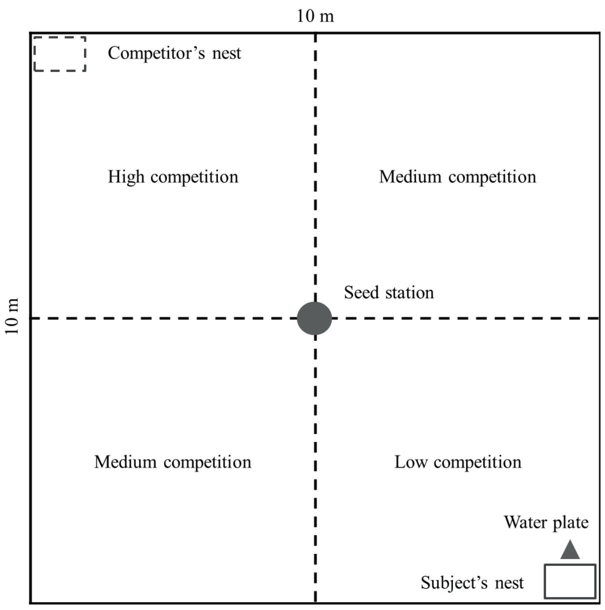

**Figure 1** Enclosure design for the experiments for seed-hoarding behavior of *Apodemus peninsulae* under the mediation of competitors.

15:00 and 16:00 hours, and kept in the enclosure for 4 days. Ten untagged seeds were provided on day 1 for environmental habituation, 30 tagged seeds were provided on day 2 for the pre-competition test, 30 tagged seeds and a competitor were provided (between 1500 and 1600 hours) on day 3 for the competition test, and, finally, the competitor was removed (between 1500 and 1600 hours) and 30 tagged seeds were provided on day 4 for the post-competition test. Water was provided *ad libitum*. Peanuts (5–10 g) were provided daily during each trial to balance the nutritional needs and starving motivation of experimental animals. In the early afternoon on each day (between 1200 and 1400 hours), animals were closed in the nest, competitors were removed (on day 3) and seed fates were recorded (see below). Enclosures were renewed by adding water and displacing seeds. At the completion of a trial, enclosures were refreshed by removing all seeds and their fragments, the nest box and water plate were replaced and the soil was loosened; a break of 12 h was allowed to limit possible interference. Experimental animals, competitors and experimental times are presented in Table 1. All experimental animals were used only once, but some competitors (3 *A. peninsulae*, 2 *T. triton* and 2 *S. davidianus*) were reused twice with at least a 7 day break.

Seed fates were recorded as: intact *in situ* (IIS), when a seed was intact and remained at the seed station; eaten (E), when a seed was consumed and the tag was left on the ground surface or in the nest; scatter-hoarded (SH), when a seed was intact and buried in the soil or grass; and larder-hoarded (LH), when a seed is intact and in the nest (also see Zhang *et al*. 2011). Total harvested seeds is the sum of E, SH and LH. The seed location of each scatter-hoarded seed was recorded as a high, medium or low competitive area (Fig. 1).

## Statistical analyses

The Shapiro–Wilk test was used to determine normality, and if $P < 0.05$, then data were $\log(x + 1)$-transformed to achieve normality. The generalized linear mixed multivariate model was used to test the main effects of competitor species, treatment and their interactions (fixed factors) on seed fates (seed number) and each competitive level of seed placement (proportion) of the experimental animals. Repeated measures ANOVA, including pairwise comparisons, was used to test for effects of experimental treatment on each seed fate and seed location area in different competitor species. Sexual effects of the experimental animals and competitors were not considered because of the small sample size. All statistical tests were 2-tailed, and differences were considered significant when $P < 0.05$. Statistical analyses were conducted in SPSS v 20.0 (SPSS, Chicago, USA).

## Results

### Seed fate

The amount of harvested seeds was significantly affected by competitor species ($\chi^2 = 123.161$, df = 3, $P < 0.001$), treatment ($\chi^2 = 147.471$, df = 2, $P < 0.001$) and their interaction ($\chi^2 = 123.146$, df = 6, $P < 0.001$). *A. peninsulae* harvested fewer seeds in the presence of *N. confucianus* ($r = 14.700$, $P < 0.001$) and *T. triton* ($r = 12.700$, $P < 0.001$), then returned to its original state of pre-competition when competitors were removed ($r = -16.700$, $P < 0.001$ for *N. confucianus*, $r = -13.200$, $P < 0.001$ for *T. triton*); whereas they did not change harvest intensity in the face of intraspecifics and *S. davidianus* but harvested more seeds when these competitors were removed ($r = -3.400$, $P = 0.033$ for intraspecifics, $r = -3.375$, $P = 0.027$ for *S. davidianus*) (Fig. 2a).

The number of eaten seeds was significantly affected by competitor species ($\chi^2 = 36.835$, df = 3, $P < 0.001$). *A. peninsulae* ate more seeds in the presence of *S. davidianus* ($r = -1.750$, $P = 0.046$), and returned to the former state after competitors were removed (Fig. 2b).

The amount of scatter-hoarded seeds was significantly affected by competitor species ($\chi^2 = 10.835$, df = 3, $P = 0.013$), treatment ($\chi^2 = 145.720$, df = 2, $P < 0.001$) and their interaction ($\chi^2 = 23.798$, df = 6, $P = 0.001$). *A. peninsulae* reduced scatter-hoarding intensity in the presence of all competitor species, and then returned to the original state of pre-competition when competitors were removed (all $P < 0.05$), with one exception that they still scatter-hoarded fewer seeds at the stage of post-competition when *N. confucianus* were competitors ($r = 2.900$, $P = 0.001$) (Fig. 2c).

The number of larder-hoarded seeds was significantly affected by treatment ($\chi^2 = 46.712$, df = 2, $P < 0.001$). The subjects increased larder-hoarding intensity in the presence of intraspecifics ($r = -6.300$, $P = 0.001$) and *S. davidianus* ($r = -4.500$, $P = 0.002$), even though these competitors were removed ($r = -3.700$, $P = 0.035$ for intraspecifics, $r = -2.875$, $P = 0.037$ for *S. davidianus*) (Fig. 2d). Larder-hoarding intensity of the experimental animals was totally restrained by *N. confucianus* ($r = 3.800$, $P = 0.002$) and *T. triton* ($r = 4.400$, $P = 0.007$), but when the competitors were removed, the subjects returned to the pre-competition state in the *T. triton* treatment ($r = -5.700$, $P = 0.001$), and larder hoarded more seeds in the *N. confucianus* treatment ($r = -4.700$, $P < 0.001$) (Fig. 2d).

Food-hoarding behavior of *A. peninsulae* varied with body size, hoarding habit (scatter *vs* larder), and dai-

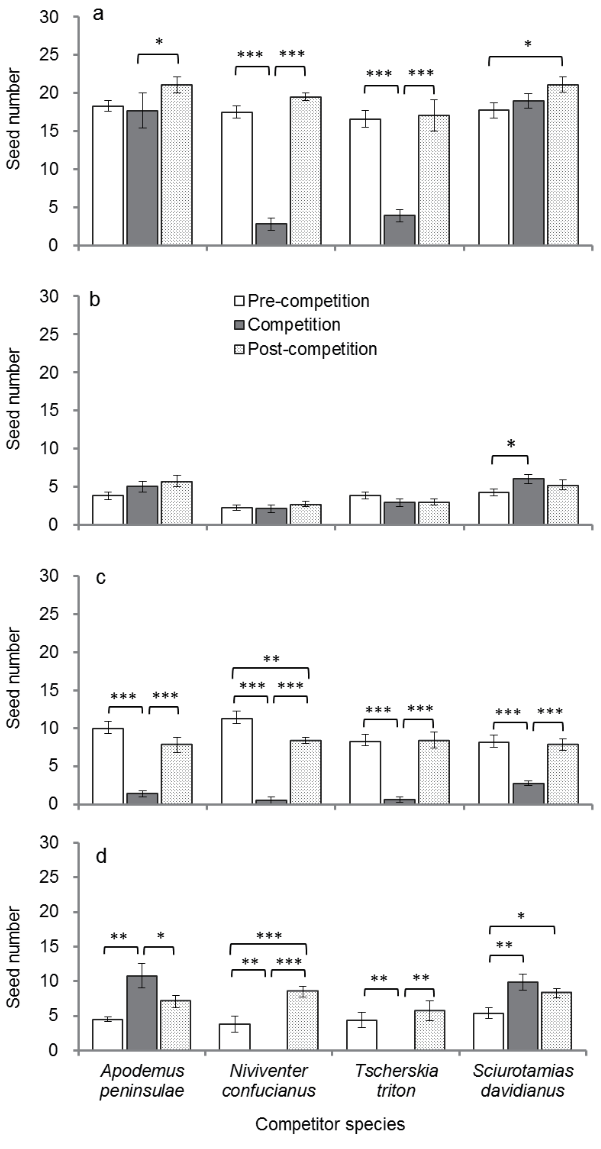

**Figure 2** Seed fates handled by *Apodemus peninsulae* in the presence of intraspecific and interspecific competitors under semi-natural enclosure conditions. Seed fates are total harvested (a), eaten (b) scatter-hoarded (c) and larder-hoarded (d). Data are mean ± SE. *$P < 0.05$; **$P < 0.01$ and ***$P < 0.001$ indicate differences between treatments.

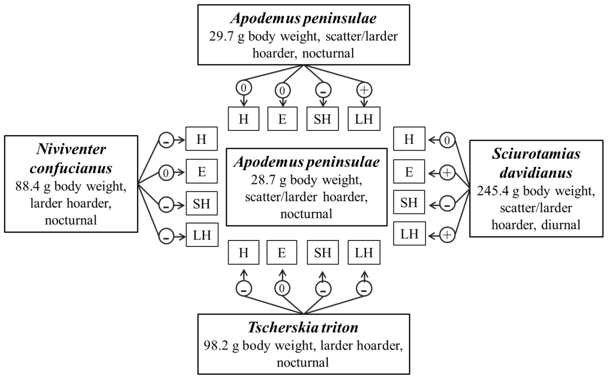

**Figure 3** Effects of intraspecific and interspecific competitors on seed-hoarding behavior of *Apodemus peninsulae* under semi-natural enclosure conditions. + is positive effects, − is negative effects and 0 is neutral effects, whereby a factor was promoted (+), suppressed (−) or not changed (0) by a competitor. Seed fates are harvested (H), eaten (E), scatter-hoarded (SH) and larder-hoarded (LH).

ly rhythm (nocturnal *vs* diurnal) of competitor species (Fig. 3). Overall, intensity of larder-hoarding was promoted, and scatter-hoarding was restrained by intraspecifics, and by *S. davidianus*, which shared similar hoarding habits (scatter and larder) but had different daily rhythms (diurnal) and body size. Hoarding intensity was totally restrained by *N. confucianus* and *T. triton*, which had large body size, larder-hoarding habit and similar daily rhythm (nocturnal). In addition, eating intensity was promoted by *S. davidianus.*

## Hoarding place

The proportion of seeds hoarded in the high competition area was significantly affected by competitor species ($\chi^2 = 8.352$, df = 3, $P = 0.039$), treatment ($\chi^2 = 74.566$, df = 2, $P < 0.001$) and their interaction ($\chi^2 = 17.720$, df = 6, $P = 0.007$). Hoarding intensity of the experimental animals in the high competition area was significantly restrained by all competitor species (All $P <$

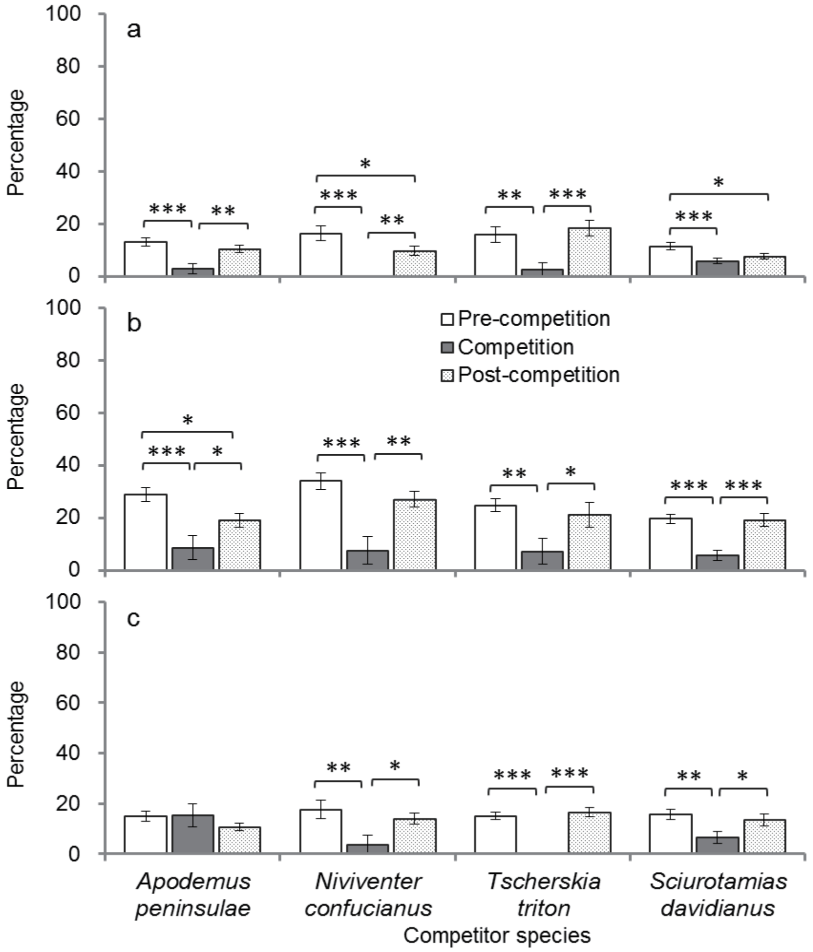

**Figure 4** Effects of intraspecific and interspecific competitors on seed-hoarding place selection of *Apodemus peninsulae* under semi-natural enclosure conditions. Cache places are high competition area (a), medium competition area (b), and low competition area (c) in the enclosure. Data are mean ± SE. *$P < 0.05$, **$P < 0.01$ and ***$P < 0.001$ indicate differences between treatments.

0.05), even though the competitors were removed in the treatments of *N. confucianus* ($r$ = 6.576, $P$ = 0.023) and *S. davidianus* ($r$ = 3.890, $P$ = 0.035) were competitors (Fig. 4a). Compared to other competitor species, *A. peninsulae* hoarded more seeds in the high competition area when *S. davidianus* were competitors.

The proportion of seeds hoarded in the medium competition area was significantly affected by competitor species ($\chi^2$ = 7.975, df = 3, $P$ < 0.047) and treatment ($\chi^2$ = 69.171, df = 2, $P$ < 0.001). The experimental animals reduced intensity of hoarding seeds in the medium competition area in the presence of all competitor species, and then returned to the original state of pre-competition after removal of competitors (all $P$ < 0.05), with an exception of the intraspecific competition treatment (Fig. 4b).

The proportion of seeds hoarded in the low competition area was significantly affected by treatment ($\chi^2$ = 69.171, df = 2, $P$ < 0.001) and the interaction between competitor species and treatment ($\chi^2$ = 22.382, df = 6, $P$ = 0.001). *A. peninsulae* reduced hoarding intensity in the low competition area when each species of interspecific competitor was present, and then returned to the state of pre-competition after competitors were removed (all $P$ < 0.05) (Fig. 4c).

Overall, seed-hoarding site selection of *A. peninsulae* was affected by competitor species, treatment and/or their interaction. Together with the results of larder-hoarding in the nests (Fig. 2d), the results showed that *A. peninsulae* reduced the intensity of scatter-hoarding outside the nest, and moved more seeds into the nest for larder-hoarding under competition from intraspecific individuals and *S. davidianus*, whereas they larder-hoarded all seeds in the nests when *N. confucianus* and *T. triton* were competitors. The subjects avoided highly competitive areas for scatter-hoarding when they faced competitors of intraspecific individuals and *S. davidianus*. In most cases, the experimental animals could return to the pre-competition state when competitive pressure was released.

## Discussion

We found that hoarding behavioral responses of *A. peninsulae* were context-dependent according to intraspecific and interspecific competitors that have varied body size, hoarding habit and/or daily rhythm but overlapped habitat utilization and diet preferences. Overall, the experimental animals increased larder-hoarding but decreased scatter-hoarding intensity in the presences of competitors that share similar hoarding habit (scatter and larder) (e.g. intraspecific and *S. davidianus*), whereas they reduced/ceased hoarding when they faced competitors that have similar daily rhythms (nocturnal), but have large body size and larder-hoarding habits (e.g. *N. confucianus* and *T. triton*) (Figs 2 and 3). *A. peninsulae* tried to avoid highly competitive areas for scatter-hoarding, and larder hoarded more seeds in the nests under the mediation of competitors (Fig. 4). Interactions of food competition were asymmetrical between *A. peninsulae* and the superior sympatric competitors (Fig. 3). Behavioral plasticity was deemed a functional response of the experimental animals to food competition because they returned to the pre-competition state in most cases when competitive pressure was released (Vander Wall & Jenkins 2003; Dally *et al.* 2006). Our results are meaningful for understanding the coexistence of sympatric rodents from the point of view of food competition in this asymmetrical system.

Our results suggest that, compared to the focal hoarders, competitors using similar means of sharing resources (e.g. between intraspecific individuals, *A. peninsulae vs S. davidianus*) have higher competitive pressure to the hoarders even though they have different active rhythms (nocturnal *vs* diurnal) (Table 1, Figs 2 and 3). It is easy to understand that intense competition exists between animals that use food items in the same manner (Zhang *et al.* 2015; Dittel *et al.* 2017; Gu *et al.* 2017). For example, intense competition exists in the granivorous rodent communities (e.g. *Tamias* spp., *Peromyscus* spp., *S. lateralis*) in eastern Sierra Nevada, USA that hoard plant seeds (e.g. *Pinus jeffreyi*, *Purshia tridentate*, *Arctostaphylos patula* and *Castanopsis sempervirens*) in fall and rely on these seeds for survival in winter (Briggs *et al.* 2009; Vander Wall *et al.* 2009; Dittel *et al.* 2017). The experimental animals increased larder-hoarding and reduced scatter-hoarding under the mediation of competitors that share similar manners of food utilization, suggesting that larder-hoarding strategy is more suitable for food protection than scatter-hoarding in *A. peninsulae* (Fig. 2c,d) (also see Zhang *et al.* 2013b). This observation is consistent with our previous studies in *A. peninsulae* when it faced competitors of intraspecific individuals (Zhang *et al.* 2011, 2014b), or when its stores were completely lost (Huang *et al.* 2011; Luo *et al.* 2014), but not when it faced competitors of *N. confucianus* (Zhang *et al.* 2011). In a study conducted in a tropical forest in southwestern China where *N. confucianus* performs both larder-hoarding and scatter-hoarding, *N. confucianus* increased larder-hoarding and shifted from scatter-hoarding to larder-hoarding when competitors (*Rattus flavipectus*) were present (Zhang *et*

*al.* 2013b). Our observations are different from those of other studies, in which animals increased scatter-hoarding intensity, spaced caches further apart or did not change hoarding strategy when exposed to competitors (Preston & Jacobs 2001; Leaver *et al.* 2007; Male & Smulders 2007). For example, presence of intraspecific individuals promoted cache spacing in *S. carolinensis* (Leaver *et al.* 2007), whereas pilfering of intraspecific individuals impelled *D. merriami* to shift from scatter-hoarding to larder-hoarding (Preston & Jacobs 2001). Larder-hoarding often makes cache defense easier for superior animals (e.g. large-size species) by preventing thieves, while scatter-hoarding generally avoids total cache loss for subordinate hoarders (e.g. small-size species) by minimizing all caches preferred by thieves at one time (Vander Wall 1990; Macdonald 1997; Preston & Jacobs 2001; Vander Wall & Jenkins 2003; Dally *et al.* 2006). Larder-hoarding is successful only if the hoarders are superior to their competitors because larder caches often suffer from high pilferage (e.g. high value, stronger odors) and need aggressive defenses (Clarkson *et al.* 1986; Dally *et al.* 2006). Scatter-hoarding, by contrast, is successful only when the hoarders can retrieve more caches to compensate for costs in terms of travel, memory and predation risks (Stapanian & Smith 1978, 1984; Dally *et al.* 2006). However, our findings are inconsistent with these predictions because the smallest *A. peninsulae* increased larder-hoarding, rather than scatter-hoarding, in the presences of competitors (also see Preston & Jacobs 2001). Several factors may determine the experimental animals' responses of larder-hoarding enhancement. First, *A. peninsulae* often hoard seeds in burrows and small caves in the field, which prevents large thieves from gaining access to these seeds due to the small size of the entrance (Table 1) (Zhang *et al.* 2014b). Second, scatter-hoarding does work in the field, but it does not work under enclosure conditions because risks of competition/pilferage and predation are very high, and/or suitable sites for scatter-hoarding are limited in the narrow and enclosed space. Our other studies conducted in a large and more complex enclosure (40 m × 50 m, which contain enough suitable cache sites and refuges) indirectly support these arguments (Zhang *et al.* 2014a; Wang *et al.* 2018; Wang 2018; Huang *et al.* 2019). In these studies, *S. davidianus* (large body and primary scatter hoarders, Table 1) rapidly hoarded seeds around seed stations and then transferred these seeds to nest areas for scatter-hoarding, rather than move these seeds into the nest for larder-hoarding (Zhang *et al.* 2014a), whereas *A. peninsulae* adopted both scatter- and larder-hoarding throughout the period of tests under the mediation of intra- and/or inter-specific competitors (Wang 2018; Huang *et al.* 2019).

Our results also show that food competition of subordinate species (small-size) was totally suppressed by superior competitors (large-size) that have overlapped habitat preference, nocturnal rhythm and diets (e.g. *N. confucianus* and *T. triton*) (Table 1, Figs 2 and 3). Inconsistent with this observation, our previous studies conducted under the similar enclosure conditions show that *A. peninsulae* individuals harvested, larder-hoarded and consumed food with great intensity when *N. confucianus* were in audience (caged, cannot compete/pilfer directly, as same as this study), and ate more of their larder-hoarded seeds and tried to transfer their scatter-hoarded caches into the nests for larder-hoarding when *N. confucianus* were pilferers (not caged, can freely pilfer) (Zhang *et al.* 2014b). This difference reflects the plasticity of hoarding behavior in *A. peninsulae*, a largely unknown phenomenon that animals can regulate hoarding strategies according to conditions of time/space/environment and risks of predation/competition/pilferage. In addition, our results suggest that there were definitely asymmetrical interactions in food competition between sympatric species that have similar ecological niches, supported by several previous studies that were conducted under enclosure conditions and in the field (e.g. (Vander Wall *et al.* 2009; Penner & Devenport 2011; Zhang *et al.* 2014b; Dittel *et al.* 2017; Gu *et al.* 2017). For example, *N. confucianus* (large-size) harvested more seeds from the seed station than *A. peninsulae* (small-size), and showed a unidirectional pilferage of seeds cached by *A. peninsulae* (Zhang *et al.* 2014b). In the pine forests (e.g. *P. jeffreyi*) of southern Reno, Nevada USA, *S. lateralis* (primary larder hoarders) were more able to forage for nuts on trees or on the ground surface, while *T. amoenus* (primary scatter hoarders) were more professional in cache-pilfering (Vander Wall *et al.* 2009). Within the asymmetrical interactions, whether or not one makes a profit is associated with its body-size, hoarding habit and competitive ability for food resources and pilferage of caches (Vander Wall *et al.* 2009; Dittel *et al.* 2017; Wang *et al.* 2018). It is interesting how a subordinate species coexists with superior competitors that shared similar ecological niches. Here, by considering interactions of seed-hoarding, we argued that context-dependent regulation of hoarding strategies according to competitors promotes subordinate species coexistence with their superior neighbors. However, this argument needs more observations in multiple species and ecosystems.

Several animals reduce foraging intensity or cease caching events to avoid further loss by competitors/pilferers (Bugnyar & Kotrschal 2002; Emery *et al.* 2004; Dally *et al.* 2006; Baudoin *et al.* 2013). Here, we argued that high competitive pressure compelled *A. peninsulae* to reduce foraging or cease caching under enclosure conditions because the subjects recovered to the original state of pre-competition in most cases when competitors were removed (Fig. 2). The observation that experimental animals avoided the high competitive areas for scatter-hoarding and moved more seeds into the nest for larder-hoarding implies that they could use alternative strategies, rather than cease foraging, to compete for resources with superior species if there were enough spaces (Fig. 4). Competition between sympatric species is less intense in the field than under enclosure conditions because there are light differentiations at micro-levels of habitat selection, rhythm, diets and hoarding behavior. At our study site, *A. peninsulae* (small-size, larder and scatter hoarder) is different from other species in micro-habitat selection, active peak time, seed preferences and/or hoarding behavior (Zhang & Zhang 2008; Zhang *et al.* 2011, 2014b; Huang *et al.* 2019). These differentiations can help *A. peninsulae* to avoid high competition and pilferage caused by superior species, and therefore promote species coexistence. Ecological niche difference at multiple levels from other species is also a contribution to prevalence of *A. peninsulae* in the study area.

Generally, *A. peninsulae* reduced/ceased foraging and hoarding, increased larder-hoarding, reduced scatter-hoarding, and/or avoided the high competitive area for hoarding according to the presence of different competitor species. Exactly which strategies were adapted should be associated with the body-size, habitat preference, diet, hoarding habit and/or daily rhythm of competitors. Together with our previous studies (e.g. Zhang *et al.* 2011, 2014b; Luo *et al.* 2014; Huang *et al.* 2019), we argued that context-dependent behavioral regulation according to competitors is beneficial for species coexistence and survival of the subordinate species in asymmetrical system. In order to demonstrate how widespread this phenomenon of behavioral plasticity occurs in this asymmetrical system, further studies are needed in multiple communities and ecosystems.

## ACKNOWLEDGMENTS

We thank F. Wang and X. Shang for assistance with fieldwork, and Jacob Wickham for English editing. This study was partially supported by the National Natural Science Foundation of China (grant no. 31772471), and the self-determined research funds of CCNU from the colleges' basic research and operation of MOE (grant no. CCNU17A02017).

**Cite this article as:**

## Cite this article as:

Niu H, Zhang J, Wang Z, Huang G, Peng C, Zhang H (2020). Context‐dependent responses of food‐hoarding to competitors in *Apodemus peninsulae*: implications for coexistence among asymmetrical species. *Integrative Zoology* **15**, 115–26.
